# Editorial: Sentience, Pain, and Anesthesia in Advanced Invertebrates

**DOI:** 10.3389/fphys.2019.01141

**Published:** 2019-09-06

**Authors:** William Winlow, Anna Di Cosmo

**Affiliations:** ^1^Department of Biology, University of Naples Federico II, Naples, Italy; ^2^Institute of Ageing and Chronic Diseases, University of Liverpool, Liverpool, United Kingdom; ^3^NPC Newton, Preston, United Kingdom

**Keywords:** sentience, pain, anesthesia, arthropods, cephalopod molluscs, pulmonate molluscs, action potentials

Sentience may be thought of as the ability to perceive events in the context of previous or future events, resulting in conscious non-reflex behavioral modification(s) and is dependent on self-awareness. The ability to perceive pain, not just nociceptive stimuli, is thus a consequence of self-awareness, which then imparts the ability to avoid potentially damaging and painful future encounters. Thus, sentience must encompass elements of both time and complexity, including learning and memory, and is dependent upon individual experiences, but at what evolutionarily convergent points in the evolution of nervous systems does the subjective sensation of pain arise? If advanced invertebrates are conscious, sentient and self-aware, then there must be numerous different neural systems with which consciousness is associated, so what sort of neural matrix is required for a creature to become sentient?

## Are Advanced Invertebrates Sentient and Can They Feel Pain?

Cephalopod molluscs and decapod crustaceans are believed to the most developed and intelligent invertebrates and may be sentient animals. They are legally protected by a number of national and trans-national organizations, such as the European Union, but it is difficult for us to understand sentience in these creatures given the major structural differences between us and them, particularly since we last shared a common ancestor about 550 million years ago (Walters).

In a theoretical article Key and Brown discuss what they describe as anecdotal evidence for sentience and sensory awareness in cephalopod molluscs. They have developed an algorithm that generates multiple levels of awareness and show that the interconnectivity of the human brain is consistent with the algorithm in generating pain awareness, but that the cephalopod brain lacks the circuitry to do this, implying that cephalopods cannot feel pain. In a separate and very extensive review, Walters considers the evolutionary nociceptive biology of a wide range of molluscs and arthropods. He points out that both molluscs and arthropods have systems that suppress nociceptive responses, developed through convergent evolution. He then considers pain-like states in cephalopod molluscs, crustaceans, and insects and points out their similarities in response to damaging or potentially damaging stimulation to the body, which implies functional awareness of injury induced vulnerability. However, he concludes that it is not yet possible to say that “any molluscs or arthropods have evolved a capacity for conscious emotion and for suffering after noxious experience.”

## Development of Anesthetic Methods for Cephalopod Molluscs

It is very difficult to assess pain in invertebrates, because a withdrawal response to a noxious stimulus could be a simple reflex reaction in simpler animals. This leads to questions as to how the more advanced invertebrates should be anesthetized. Two differing approaches have been outlined in this Research Topic. Butler-Struben et al. have carried out *in vivo* recording of neural and behavioral correlates of anesthesia in cephalopods and conclude that magnesium chloride and ethanol are suitable anesthetic agents for these animals. In their review Winlow et al. conclude that anesthesia in *Octopus Vulgaris* is best achieved with clinical anesthetics, but agree that pre-treatment with a muscle relaxant such as magnesium chloride, acting as an anesthetic adjuvant, might be the best future approach. Much of the work on clinical anesthetic techniques has been developed using simpler molluscs such as the pond snail *Lymnaea stagnalis* as outlined by Moghadam et al., who have discovered significant differences between the responses of identified motor neurons and interneurons to both applied systemic and volatile anesthetics, *in situ* and in single cell culture.

## Usefulness of Cell Culture Techniques

Although general anesthetics are considered to be safe and effective, care must be taken in their use as they can have cytotoxic effects particularly during peak periods of neurodevelopment. Armstrong et al. have reviewed this issue and have demonstrated the usefulness of synaptically connected identified neurons from *Lymnaea* when grown in cell culture. They also demonstrated some novel preliminary data on the newer anesthetic agent dexmedetomidine on synaptic transmission using these techniques. Such techniques have proved difficult to apply to cephalopods, which have much smaller neurons than do pulmonate molluscs such as *Lymnaea*. However, this problems is being resolved by the studies of Maselli et al. and Maselli et al., who have now developed suitable techniques for primary cell culture of dissociated neurons from two specific brain regions of *Octopus vulgaris*, the vertical superior frontal system and the optic lobes. These regions of the brain are involved in memory, learning, sensory integration, and adult neurogenesis. The data obtained from this work opens the prospect of more detailed studies of injury-induced neuronal regeneration in *Octopus* brain regions, which may be equivalent to those of vertebrates.

## Getting the Basics Right

One of the key points in our understanding of the functioning of nervous systems in general was the discovery of the ionic mechanisms underlying the action potential using the squid giant axon by Hodgkin and Huxley (1952). However, Johnson and Winlow indicate that this is by no means the whole story. More recent findings suggest that action potentials are accompanied by a soliton pressure wave which may instigate channel opening. What is more, it is suggested that that the action potential should be considered as a ternary rather than a binary event, thus including the refractory period in computational models of interactions between colliding action potentials. If action potentials are not yet fully understood then one of the key elements in understanding sentience is imperfect, with potential negative consequences for our understanding of sentience and also for the development of artificial intelligence.

## Author Contributions

Both authors listed have made a substantial, direct and intellectual contribution to the work, and approved it for publication.

### Conflict of Interest Statement

The authors declare that the research was conducted in the absence of any commercial or financial relationships that could be construed as a potential conflict of interest.
